# Expression of stromal elements of prostatic adenocarcinoma in different gleason scores^1^

**DOI:** 10.1590/s0102-865020190100000005

**Published:** 2019-12-13

**Authors:** Clarice Fraga Esteves Maciel Osorio, Waldemar Silva Costa, Carla Braga Mano Gallo, Francisco José Barcellos Sampaio

**Affiliations:** I Fellow PhD degree, Postgraduate Program in Physiopathology and Surgical Sciences, Urogenital Research Unit, Universidade do Estado do Rio de Janeiro (UERJ), Brazil. Conception and design of the study; acquisition, analysis and interpretation of data; technical procedures; histological examinations; statistics analysis; manuscript preparation and writing; final approval; II PhD, Associate Professor, Urogenital Research Unit, UERJ, Rio de Janeiro-RJ, Brazil. Conception and design of the study, technical procedures, histological examination, interpretation of data, manuscript preparation and writing, final approval; III PhD, Researcher, Urogenital Research Unit, Rio de Janeiro-RJ, Brazil. Conception and design of the study, interpretation of data, statistics analysis, manuscript preparation and writing, final approval; IV PhD, Full Professor, Urogenital Research Unit, UERJ, Rio de Janeiro-RJ, Brazil. Conception and design of the study, interpretation of data, critical revision, final approval

**Keywords:** Prostatic Neoplasms, Neoplasm Grading, Stromal Cells, Histology

## Abstract

**Purpose:**

To quantify and compare the expression of stromal elements in prostate adenocarcinoma of different Gleason scores with non-tumor area (control).

**Methods:**

We obtained 132 specimens from samples of prostate peripheral and transition zone. We analyzed the following elements of the extracellular matrix: collagen fibers, elastic system, smooth muscle fibers and blood vessels. The tumor area and non-tumor area (control) of the TMA (tissue microarray) were photographed and analyzed using the ImageJ software.

**Results:**

The comparison between the tumor area and the non-tumor area showed significant differences between stromal prostate elements. There was an increase of collagen fibers in the tumor area, mainly in Gleason 7. Elastic system fibers showed similar result, also from the Gleason 7. Blood vessels showed a significant increase occurred in all analyzed groups. The muscle fibers exhibited a different behavior, with a decrease in relation to the tumor area.

**Conclusions:**

There is a significant difference between the extracellular matrix in prostate cancer compared to the non-tumor area (control) especially in Gleason 7. Important modifications of the prostatic stromal elements strongly correlate with different Gleason scores and can contribute to predict the pathological staging of prostate cancer.

## Introduction

During the past decade, various methods have been used to determine the prognosis of patients with prostate cancer (PC). Among them, is the histopathological classification proposed by Gleason^1^.

This classification has undergone some modifications, but is still widely used because the correlation between the Gleason score and mortality is very significant^2^ ^,^ ^3^. A Gleason score of 6 is low grade cancer, 7 is intermediate grade, and a score of 8 to 10 is high grade cancer. Patients with low grade adenocarcinoma almost never develop aggressive disease, while those with high grade (Gleason score 8 to 10), in most cases, die of PC^4^.

The combination of clinical staging and Gleason score is still the best predictor of prognosis^4^ ^,^ ^5^. Currently, the prognostic factors established for PC are ‘TNM Classification of Malignant Tumors’, the surgical margin status, the serum level of PSA (prostate specific antigen) and Gleason's score^6^ ^–^ ^9^. Even though the Gleason classification is the most commonly used, it remains insufficient to clarify the tumor behavior^2^, also it cannot be applied in some histopathological variants, e.g. small cell carcinoma, squamous cell carcinoma, transitional cell carcinoma and basaloid/adenoid cystic carcinoma^4^.

The prostate gland is composed of epithelial and stromal compartments. Similarly, the PC is composed of malignant epithelial cells and the stroma, whose transformation is important for tumor growth and development^10^.

The prostatic stroma is composed of fibrous elements of the extracellular matrix (ECM): collagen, elastic system fibers, smooth muscle fibers, fibroblasts, myofibroblasts, blood vessels, nerves and amorphous ground substance consisting of proteoglycans and glycosaminoglycans. These are the most important elements in the growth and differentiation of the normal prostate, benign prostatic hypertrophy (BPH) and PC^11^. The characteristics of stromal components in addition to their expression in the prostate tissue appear to correlate with the location of PC in the peripheral zone^12^ ^,^ ^13^.

The classification of PC into Gleason score is a well established indicator that has endured the test of time^8^. However, it is a subjective method and based exclusively on the characteristics of parenchyma. The evaluation and algorithm classification is based on two fundamental criteria: the degree of glandular differentiation and tumor development architectural pattern^14^.

A quantitative analysis of the elements that constitute the stroma of PC, associated with the prognostic features and the Gleason score, shows a correlation in the progression and metastasis, and can contribute to new prognostic approaches^14^ ^,^ ^15^.

The goal of this work was to quantify and correlate changes in the prostate stromal elements with different Gleason scores in adenocarcinoma with the non-tumor area (control), as Gleason's histopathological classification takes into account only the parenchyma.

## Methods

This project was approved by the Ethics Committee (CAAE number 12685413.6.0000.5259) – Universidade do Estado do Rio de Janeiro, Brazil.

We retrospectively analyzed a total of 132 samples obtained from open radical prostatectomies. The procedures were performed at private hospitals in the city of Rio de Janeiro, Brazil. We compared the tumor area with the non-tumor (control) area of the same patient to analyzed samples with the same genome. The specimens were obtained from samples of prostate peripheral and transition zone.

The mean age of the patients was 63 years old (ranging from 45 to 82 years old).

We did not include prostate acinar carcinoma with neoadjuvant treatment. We also did not include in this work the following carcinomas: prostate ductal carcinoma, small cell carcinoma, squamous cell carcinoma, transitional cell carcinoma and basaloid/adenoid cystic carcinoma.

Once surgically removed, the samples were sent to the Pathological Laboratory and fixed in formaldehyde (4%) for 24 to 48 hours. Surgical margins were stained with India ink and sectioned into eight quarters, from which samples were taken for histological analysis. We also removed fragments of the vesical and urethral margins.

The samples were placed in cassettes for routine processing and embedded in paraffin. Histological sections of 5-µm were obtained from each block and mounted on slides.

The sections were stained using different methods. Histochemical methods: Hematoxylin and Eosin, Masson's trichrome and the Weigert's method for staining elastic system fibers. For immunohistochemical analysis for staining blood vessels, we used the antibody CD 31 (Abcam, policlonal, Ref: ab28364, Cambridge, USA).

The slides were observed with a light microscope Nikon binocular YS100. The slides confirmed the diagnosis of prostatic adenocarcinoma, using the Gleason classification. The samples studied were separated into three groups according to the Gleason score: Gleason 6 (n = 44), Gleason 7 (n = 64) and Gleason 8 to 10 (n = 24). The group of Gleason 7 was subdivided into two subgroups: 3 + 4 and 4 + 3.

Both the tumor area and the non-tumor area (control) were selected and marked from paraffin blocks (donor's blocks). It was stipulated that the area marked in blue corresponded to adenocarcinoma and the area marked in red corresponded to the non-tumor area (control).

A fragment was collected from each selected area of the donor's block by direct puncture using a 1 mm needle. These fragments were included in a new block (receiver's block). This block received up to 304 fragments (16 columns versus 19 lines) oriented to indicate the origin of each of them. Tissue microarray (TMA) histological sections were obtained and sectioned at 5-µm thickness.

The following fibrous elements were analyzed from ECM: collagen, elastic system fibers, smooth muscle fibers, and blood vessels.

All areas, tumor and non-tumor (control), of the TMA slides were photographed with an X200 objective with a digital camera (DP70) attached to a microscope Olympus BX51, Tokyo, Japan. The images were captured and analyzed using ImageJ software 1.46 (National Institute of Health, Bethesda, USA).

Statistical analysis was calculated using an unpaired “t” test and the program Graph Pad Prism 5.03 version for Windows (Graph Pad Software, San Diego, California, USA). The differences were considered statistically significant when p < 0.05.

## Results

**Figure 1:**
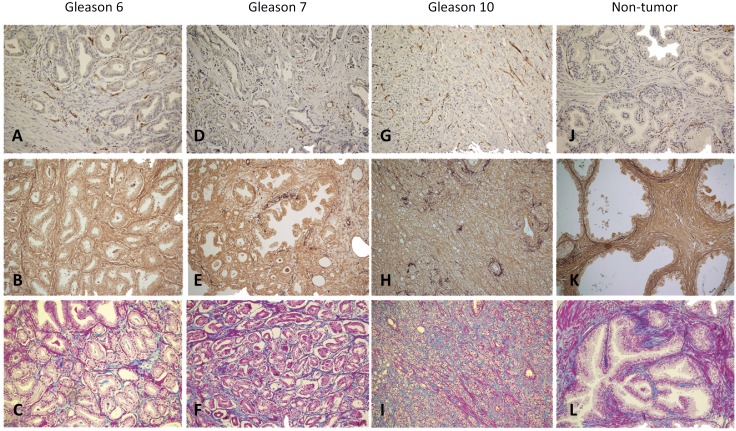
Photomicrographs of blood vessels in tumor areas Gleason 6 **(A)**, Gleason 7 **(D)**, Gleason 10 **(G)** and non-tumor areas **(J)**, CD31, x200. Elastic system fibers in tumor areas Gleason 6 **(B)**, Gleason 7 **(E)**, Gleason 10 **(H)** and non-tumor areas (K), Weigert, x200. Collagen fibers in tumor areas Gleason 6 **(C)**, Gleason 7 **(F)**, Gleason 10 **(I)** and non-tumor areas **(L)**, Masson trichrome, x200.

**Figure 2:**
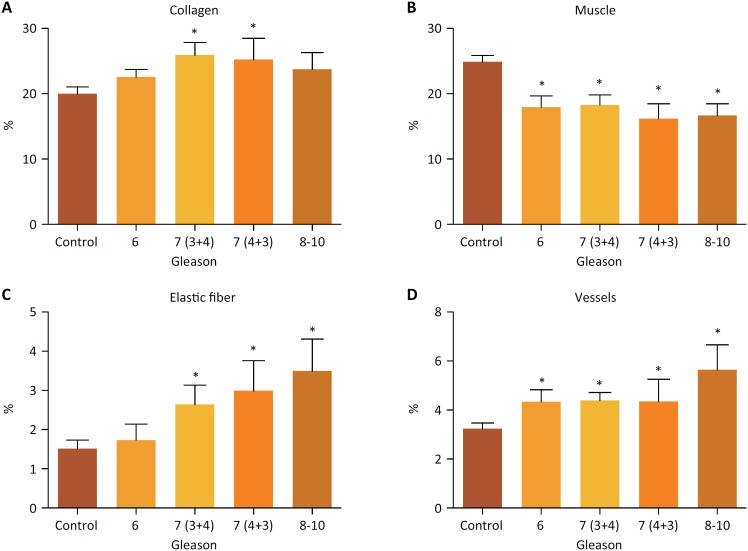
**A.** Quantitative analysis of collagen fibers in the non-tumor group (control), in Gleason 6 group, in Gleason 7 (3 + 4) and Gleason 7 (4 + 3) groups, and in Gleason 8-10 group. **B.** Quantitative analysis of smooth muscle fibers in the non-tumor group (control), in Gleason 6 group, in Gleason 7 (3 + 4) and Gleason 7 (4 + 3) groups, and in Gleason 8-10 group. **C.** Quantitative analysis of elastic system fibers in the non-tumor group (control), in Gleason 6 group, in Gleason 7 (3 + 4) and Gleason 7 (4 + 3) groups, and in Gleason 8-10 group. **D.** Quantitative analysis of vessels in the non-tumor group (control), in Gleason 6 group, in Gleason 7 (3 + 4) and Gleason 7 (4 + 3) groups, and in Gleason 8-10 group. (*)Statistically significant difference.

Results are presented on Table 1, and Figures 1 and 2.

**Table 1:** Quantitative analysis of prostatic stromal elements in non-tumor and tumor areas associated with Gleason score and subtypes of Gleason 7.

	Non-tumor(control)	Gleason 6n=44M age=62.70	Gleason 7 (3+4)n=45M age=64.20	Gleason 7 (4+3)n=19M age=64.20	Gleason 8-10n=24M age=64.99
Collagen (%)	19.90	22.46	26*	25.70*	23.74
Elastic fiber (%)	1.48	1.81	2.54*	3.05*	4.08*
Muscle (%)	24.87	17.08*	18*	16*	15.78*
Vessels (%)	3.22	4.39*	4*	4.33*	5.66*

* Statistical difference between the non-tumor group (control) and the Gleason 6, Gleason 7 (3 + 4), Gleason 7 (4 + 3) and Gleason 8-10 groups. M age = mean age.

The comparison between the tumor and non-tumor area (control) of the same patient showed significant differences between the prostatic stromal elements.

Statistical analysis showed differences in the stroma, between the tumor area and the non-tumor area (control), in all Gleason scores.

The group of Gleason score 6, when compared to the non-tumor group (control), showed no statistical difference for collagen and elastic system fibers. There was a decrease in muscle fibers in the Gleason 6 group when compared to the non-tumor group (control). In contrast, there was an increase of vessels in the Gleason 6 group when compared to the non-tumor group (control).

The Gleason 7 group, when compared to the non-tumor group (control), showed statistical difference for all parameters analyzed.

The Gleason 7 (3+4) group, when compared to the non-tumor group (control), showed increased of collagen fibers, elastic system fibers, and blood vessels. On the other hand, it showed a decrease of smooth muscle fibers.

The Gleason 7 (4+3) group, when compared to the non-tumor group (control), showed similar results to the Gleason 3+4 group.

However, if we compare the two Gleason 7 groups, we observed that there is difference between them (Table 1). These differences at stromal level further justify the division of Gleason 7 in two subgroups (4+3 and 3+4), as proposed by Gleason.

The Gleason group 8-10, when compared to the non-tumor group (control), showed statistical difference for the elements analyzed, except for collagen fibers. There was a statistical increase in elastic system fibers and vessels in the Gleason group 8-10 when compared to the non-tumor group (control). In contrast, there was a decrease in smooth muscle fibers in the Gleason 8 group when compared to the non-tumor group (control).

## Discussion

In a previous article, one of the authors of the present study showed a correlation between the primary Gleason score and nuclear medium volume^17^. However, PC is not only a disease of abnormal epithelial cell proliferation, but a disease that affects the complex interactions between prostatic epithelial cells and stromal compartment^18^. Histopathological differences observed in varying Gleason scores, showed that there is a constant change in the stromal of PC. The development of aggressive neoplasm appears to be associated with the biosynthesis of the ECM and, therefore, with changes in its structure^15^ ^,^ ^19^. Neoplastic epithelial cells, in interaction with stromal cells, and other elements of the ECM, create a microenvironment susceptible to proliferation and differentiation in carcinogenesis^8^.

Quantitative characterization of stromal parameters plus the parenchymal features determined by the Gleason classification demonstrates an association between them and corroborates the prognosis of PC. In 1994, Nakada and Kubota^19^ showed that the concentration of collagen and non-collagen proteins was similar both in BPH and PC. In this study, there was an increase of the collagen in the tumor area in relation to the non-tumor area. We observed a statistical increase in Gleason 7 of approximately 30%. The results are in agreement with Zhang *et al*.^12^ that point out an increase of collagen fibers in the PC and a decrease of muscle fibers.

According to Cunha *et al*.^20^ the PC involves a sequential disruption in the interaction of epithelium-smooth muscle, resulting in a vicious cycle of progressive dedifferentiation of both the epithelial component as smooth muscle, which would lead to tumor progression. A decrease in muscle fibers corroborates the hypothesis that the modified epithelium is unable to maintain normal adjacent muscle differentiation^21^. In this study we observed a reduction of smooth muscle fibers in all Gleason scores analyzed. The findings of Wong and Tam^21^ also support this.

There is little data on the role of elastic system fibers and their receptors in tumor invasion. It is known that there is intra tumor disorganization of elastic fibers in the stroma of PC^10^. In addition to this disorganization, our results showed that they have suffered an increase as the Gleason score increases. This increase was significant in Gleason 7 and Gleason 8-10. The concentration of elastic fibers in well and moderately differentiated PC showed to be greater than in the BPH^19^, which was also observed in this study.

Microvessel density is considered an important prognostic factor and therapeutic target in several types of tumors, such as breast cancer, colon, cervix, melanoma and carcinoma of the head and neck, but its meaning in PC is still controversial^22^. However, the mechanism and regulation control of angiogenesis is of great importance for the design of new strategies in the treatment of PC. According to a study by Bono *et al*.^23^, there is an association of high Gleason score with high density of microvessels. Other authors^24^ ^,^ ^25^ have showed this same observation. Previous studies have shown increased angiogenesis in PC and correlation with tumor score, stage, progression and survival. However, subsequent studies have failed to confirm a prognostic value in microvessel density. Our results showed a significant increase of blood vessels according to the Gleason score, which is consistent with the first studies that showed an increase in PC angiogenesis^23^. Although previous studies showed that the density of microvessels is not yet a prognostic parameter^22^, this study provides more data that along with data from prior studies can corroborate the prognosis of PC. Studies of different types of cancer in humans showed stromal cells activated phenotypes, could induce composition change from MEC and increase the density of microvessels^26^.

The microenvironment in which such tumor cells develop into an aggressive phenotype is highly heterogeneous. The interruption in the communication between stroma and parenchyma could lead to the development of anti-cancer therapies targeting the tumor stromal elements^18^. Our work sought to analyze the behavior of such elements in the different Gleason scores.

## Conclusions

Our data show that significant modifications of the prostatic stromal elements strongly correlate with different Gleason scores, especially for the Gleason 7 or higher, and can contribute to predicting pathological staging of prostate cancer. Also, these data can contribute for the studies of the morphological substrate of prostate cancer.
